# Quadriceps Tendon Rupture in an Adolescent Athlete

**DOI:** 10.1155/2017/2718013

**Published:** 2017-09-26

**Authors:** William A. Zuke, Beatrice Go, Alexander E. Weber, Brian Forsythe

**Affiliations:** ^1^Department of Orthopedic Surgery, Rush University Medical Center, Chicago, IL 60612, USA; ^2^Department of Orthopaedic Surgery, Keck School of Medicine of University of Southern California, Los Angeles, CA 90033, USA

## Abstract

Quadriceps tendon rupture is an uncommon injury that usually occurs in middle-aged and elderly men with a history of chronic illnesses. We report the case of a 17-year-old healthy adolescent male baseball player who sustained this injury as a result of sudden deceleration in his left knee. He initially reported to the emergency department and then presented to our practice, where he was diagnosed with a quadriceps tendon tear. Preoperatively, he was unable to perform a single straight-leg raise. During surgical repair, the free edge of the quadriceps tendon was mobilized and anchored into the superior pole of the patella, followed by sutures to ensure reinforcement of the quadriceps footprint. His postoperative course progressed normally, demonstrating full range of motion at 3 months and return to team training at 5 months. Unlike previously reported quadriceps tendon ruptures in adolescents, to our knowledge, this is the first report of a patient who sustained such an injury without previous trauma to his knee or quadriceps mechanism. It is possible that weakened tendon integrity from repeated microtrauma during training combined with the sudden weight change distribution may have resulted in the injury. As urgent surgical intervention is necessary to ensure efficient recovery and return to sport, the sports medicine practitioner must remain educated and vigilant on caring for these patients.

## 1. Introduction

Quadriceps tendon ruptures (QTRs) are relatively uncommon injuries (1.37/100,000) that usually present in middle-aged and elderly men with a history of chronic illness, such as diabetes, renal failure, gout, rheumatoid arthritis, or hyperparathyroidism [1, 2]. Resulting from either direct or indirect trauma to the leg, this condition usually presents unilaterally, although bilateral spontaneous injuries have been reported in patients with preexisting obesity [3]. The general recommendation is to perform surgical intervention within 48–72 hours after injury, as conservative treatment for full tears leads to poor functional outcomes [4]. Delayed repair can also lead to further complications, such as intraoperative tendon retraction and postoperative stiffness.

QTRs in children and adolescents are exceedingly rare injuries, with sparse literature reporting findings within this patient population [5–7]. The few publications that describe adolescent QTRs suggest that the injuries result from direct trauma rather than the sequelae from preexisting conditions. Additionally, QTRs in adolescents are reportedly associated with a history of previous minor trauma, compromising tendon integrity. This case report serves two objectives: first, to present the history, diagnosis, and treatment of a 17-year-old male baseball player who sustained a quadriceps tendon rupture secondary to an acute deceleration, in pursuit of a ball hit into the outfield, and second, to highlight a novel presentation of a QTR, since early intervention supports better outcomes. The sports medicine physician should thus maintain a general awareness of this uncommon injury in the young athlete.

## 2. Case Report

The authors have obtained the patient's informed written consent for print and electronic publication of the case report. The patient is a 17-year-old male (BMI 24.4) with no significant prior surgical or medical history. He initially presented to the emergency department for evaluation, consultation, and management of left knee pain. The injury occurred while he was playing in the outfield for his high school baseball team. After a sudden deceleration, he felt immediate pain in the suprapatellar region of his left knee, which led to immediate transport to the emergency department. He presented to us with an immobilizer brace and crutches. He denied any antecedent events and had been exercising regularly for the upcoming baseball season.

Physical examination of the knee revealed passive range of motion from 20 degrees of flexion to 110 degrees of flexion. A severe effusion was present, and he was tender to palpation along his anterior, lateral patella. A palpable defect was appreciated at the superolateral pole of the patella. He was unable to perform single straight-leg raise. He maintained full range of motion of the hip and ankle. Strength testing was deferred due to severe pain. An aspiration was notable for 20 mL of bloody fluid, confirming the presence of a hematoma.

**Figure 1:**
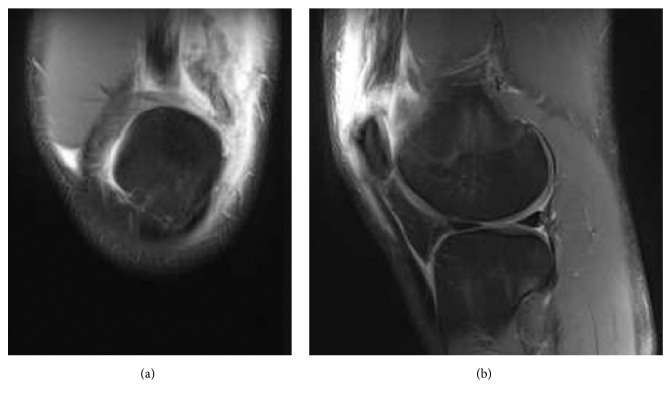
Coronal (a) and sagittal (b) representative magnetic resonance imaging (MRI) images denoting a quadriceps rupture in an otherwise healthy 17-year-old competitive athlete.

Review of the plain radiographs from the emergency department was negative for fractures or loose bodies and demonstrated normal patellar height. A subsequent MRI was obtained to further investigate the injury. The imaging revealed a full-thickness quadriceps tendon tear, predominantly along the lateral aspect, with an associated focal tear of the vastus lateralis muscle (Figure 1). Other than a grade 1 sprain of the fibular collateral ligament, no other injuries were noted. These findings were consistent with quadriceps tendon rupture, and he was scheduled to have surgery the following day.

**Figure 2:**
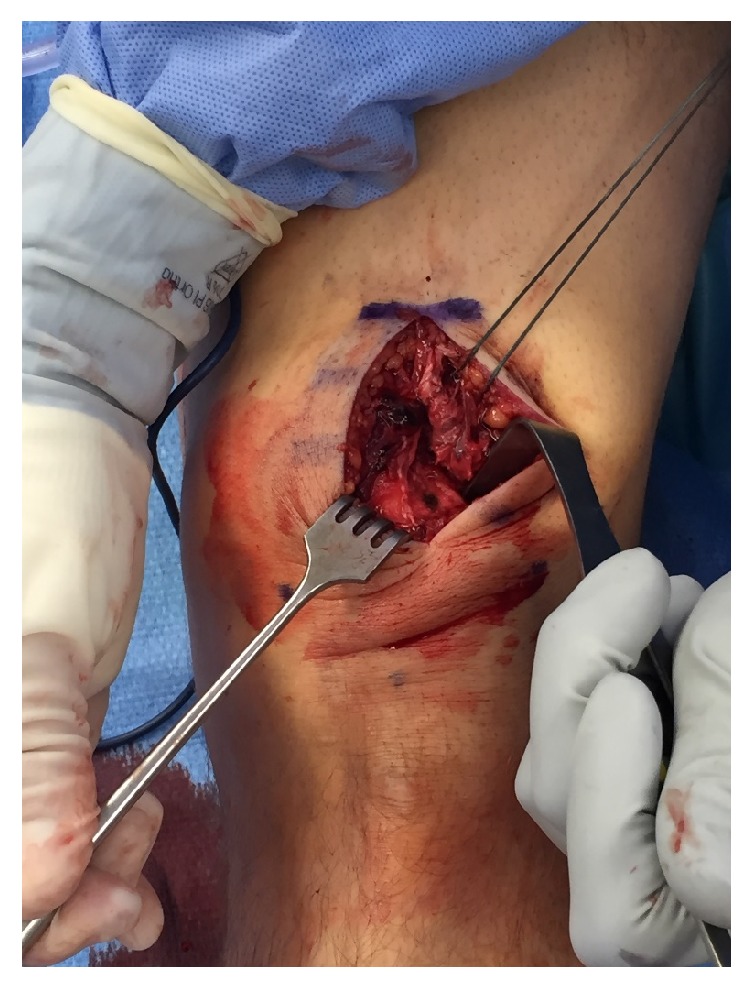
Intraoperative picture of the full-thickness quadriceps tear. Incision is slightly lateral to midline.

**Figure 3:**
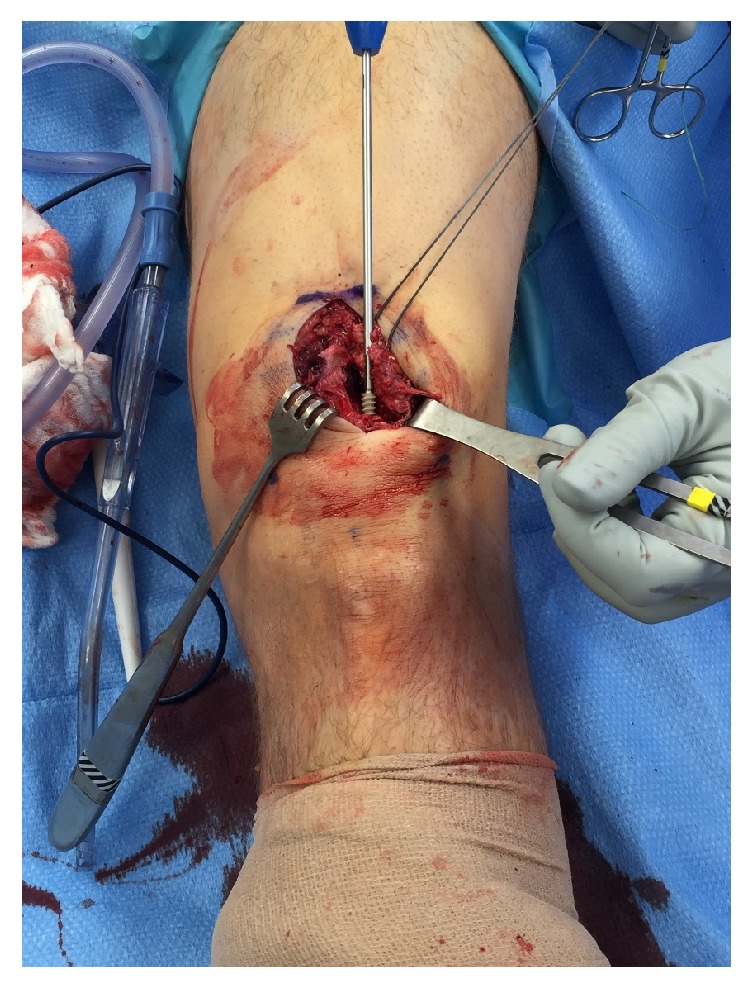
Anchor being placed in the superolateral quadriceps tendon footprint of the proximal pole of the patella.

**Figure 4:**
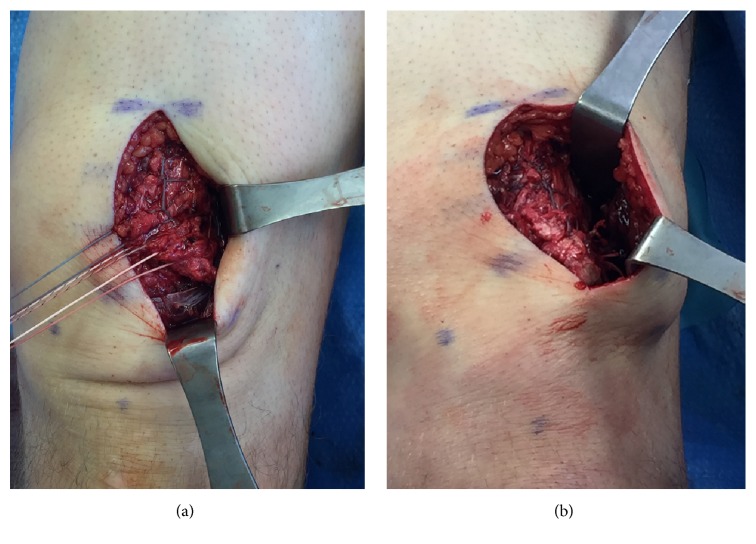
(a) Sequential Krackow suturing of the tendon tear with the anchor suture. (b) The final repair construct including the Krackow stitches of the anchor sewn into the patella periosteum.

Repair was performed through a longitudinal midline incision of 6 cm, beginning at the superior pole of the patella and extending proximally. The quadriceps was visualized, revealing a full-thickness rupture of the rectus femoris, vastus intermedius, and vastus lateralis tendon at the bone-tendon junction (Figure 2). The laterally based tear of the quadriceps was mobilized and controlled with a 0-Ethibond whipped traction stitch (Ethicon, Cincinnati, Ohio). The superior pole of the patella along the quadriceps insertion was predrilled with an 11/64 inch drill bit, and a triple loaded 5.5 PEEK corkscrew suture anchor (Arthrex, Naples, Florida) was placed (Figure 3). The tendon footprint on the patella was preserved for this repair. Three running Krackow sutures (one medial and two lateral) were placed and anchored on the distal quadriceps tendon to reinforce and ensure complete coverage of the quadriceps tendon footprint (Figure 4). The final repair construct reapproximated the torn tendon edge to the superolateral aspect of the superior pole of the patella (Figure 4).

**Figure 5:**
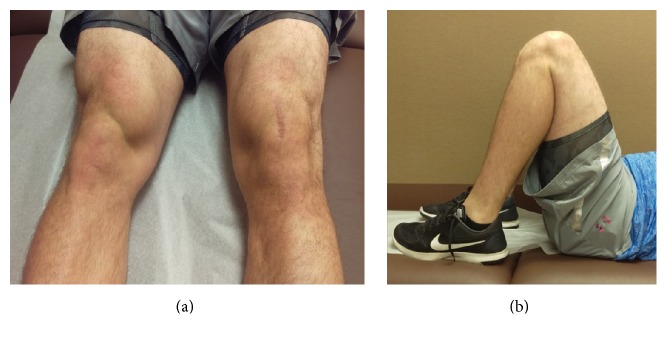
Primary healed scar without sings of infection with full knee extension (a) and full knee flexion (b) at six months after operative visit.

The patient's postoperative course was uneventful. Postoperatively he was fitted with a locking hinged knee brace (Breg, Carlsbad, California), which was worn for 6 weeks locked in extension when standing. He initiated a continuous passive motion machine on postoperative day one, starting at 30 degrees. ROM was permitted to increase 10 degrees per day as tolerated to achieve 90 degrees within 2 to 4 weeks. From 4 to 8 weeks, ROM was permitted to graduate to full flexion. Toe touch weight bearing with the knee brace locked in extension was allowed the first week, followed by weight bearing as tolerated with the knee brace locked straight. At six weeks, physical examination revealed no effusion, a ten-degree flexion deficit versus the contralateral knee, and he was able to complete a straight-leg raise without a lag. At three months postoperatively, he had regained full range of motion. At four months, he had progressed to sprinting, albeit with some difficulty. At five months he was sprinting normally and squatting 135 lbs. He returned to full baseball training with his team and continued with his home exercise regimen. At six months, he reported a maximum score of 16 on the Marx Activity Rating Scale and 93.1 on the International Knee Documentation Committee (IKDC) score [8] and a score of 75 on the Knee injury and Osteoarthritis Outcome Score (KOOS) Sports and Recreational Activities. Regarding the KOOS daily living, pain, quality of life, and symptoms, the scores at six months were 100, 100, 87.5, and 89.3, respectively. Physical exam demonstrated good quadriceps activation, with appreciable muscle atrophy, and full range of motion (Figure 5). At 1 year, he has completely returned to baseball, noting no functional deficits. His 1-year Marx Activity Rating remains unchanged, and at 1 year, the IKDC score was 87.36. KOOS sports and recreational activities, daily living, pain, quality of life, and symptoms at 1 year were 65, 100, 83.33, 81.25, and 89.29, respectively. He remains a division 1 collegiate prospect.

## 3. Discussion

Rupture of the quadriceps tendon typically occurs in males over 40 years of age, particularly in those with chronic conditions that degenerate the intratendinous structure of the knee [9]. However, there are few publications that describe this type of injury in the adolescent. Alexander et al. reported on a 15-year-old boy who initially sustained a traumatic patellar subluxation while playing basketball [6]. Repeated heavy stress on the knee before complete rehabilitation resulted in a complete quadriceps mechanism rupture, which was repaired with suture anchors along with a lateral patellar retinacular release. Adolphson reported on a traumatic quadriceps tendon rupture case in a 16-year-old girl who sustained an intramuscular hematoma on her left knee [5]. After initial rehabilitation, direct impact on her left knee resulted in a quadriceps tendon rupture, with an avulsion fracture of the patella.

Interestingly, this patient had no prior history of injury to his knee or quadriceps mechanism, which is unlike the few case reports in the literature. The cases outlined above all report a previous insult to the quadriceps mechanism or involve a premature advancement in activity level, which may have predisposed the tendon to rupture. Matsumoto et al. described a partial QTR in a 10-year-old boy without a history of trauma, but this was a case of persistent knee pain for nearly 3 years, not an acute rupture in a healthy adolescent [10].

To our knowledge, this is the first report in the literature of an acute, full-thickness quadriceps tendon rupture in an adolescent in the absence of prior injury. This case is also unique in that it does not involve a mechanical fall or impact. This condition is rare in adolescents due to the strength of the muscle tendon bone complex. Furthermore, without a previous insult to the knee, this type of injury is quite unexpected in the adolescent population. A possible contributing factor could involve repetitive microtrauma that compromised tendon integrity and thus predisposed the tendon to rupture, which could certainly be the case in a young, determined athlete who overtrained without sufficient recovery [7]. Alternatively, the traumatic injury could have been a result of a sudden change in weight distribution in combination with an eccentric quadriceps contraction resulting in enough force to tear the tendon.

In conclusion, this is a very unusual case of an acute, full-thickness quadriceps tendon rupture in a healthy adolescent athlete without a history of antecedent trauma or prodromal symptoms. It has been generally assumed that healthy tendons do not rupture, which makes this case a unique addition to the literature. Because this injury pattern demands urgent treatment, the sports medicine physician must maintain a familiarity and general awareness of this diagnosis.
